# An ecologically constrained procedure for sensitivity analysis of Artificial Neural Networks and other empirical models

**DOI:** 10.1371/journal.pone.0211445

**Published:** 2019-01-30

**Authors:** Simone Franceschini, Lorenzo Tancioni, Massimo Lorenzoni, Francesco Mattei, Michele Scardi

**Affiliations:** 1 Department of Biology, University of Rome Tor Vergata, Rome, Italy; 2 Department of Chemistry, Biology and Biotechnology, Perugia, Italy; University Of Bristol, UNITED KINGDOM

## Abstract

Sensitivity analysis applied to Artificial Neural Networks (ANNs) as well as to other types of empirical ecological models allows assessing the importance of environmental predictive variables in affecting species distribution or other target variables. However, approaches that only consider values of the environmental variables that are likely to be observed in real-world conditions, given the underlying ecological relationships with other variables, have not yet been proposed. Here, a constrained sensitivity analysis procedure is presented, which evaluates the importance of the environmental variables considering only their plausible changes, thereby exploring only ecological meaningful scenarios. To demonstrate the procedure, we applied it to an ANN model predicting fish species richness, as identifying relationships between environmental variables and fish species occurrence in river ecosystems is a recurring topic in freshwater ecology. Results showed that several environmental variables played a less relevant role in driving the model output when that sensitivity analysis allowed them to vary only within an ecologically meaningful range of values, i.e. avoiding values that the model would never handle in its practical applications. By comparing percent changes in MSE between constrained and unconstrained sensitivity analysis, the relative importance of environmental variables was found to be different, with habitat descriptors and urbanization factors that played a more relevant role according to the constrained procedure. The ecologically constrained procedure can be applied to any sensitivity analysis method for ANNs, but obviously it can also be applied to other types of empirical ecological models.

## 1. Introduction

Fish assemblage diversity in freshwater ecosystems constitutes a valuable natural resource in economic, scientific, cultural and educational terms [1]. Its conservation and management face threats as overexploitation of inland waters, flow modification, water pollution, habitat degradation and invasion by exotic species [2], [3]. Identifying the relationships between fish species richness and habitat complexity at a local scale is one of the primary concerns in understanding how environmental descriptors actually affect fish biodiversity [4], [5], [6].

In this respect, the ecological variables that can be taken into account are often characterized by complex and non-linear dependencies [7]. Ecological models have been increasingly applied in the management and conservation of freshwater fish communities, especially to predict spatial patterns of fish occurrence [8], [9]. In particular, Artificial Neural Networks (ANNs) modeling has proved to be a valuable method in order to assess whether predictable relationship between environmental descriptors and fish species richness exist in small stream environments [10], [11], [12].

While in the past ANNs were defined as “black boxes” since the computational processes taking place inside them are not easy to untangle, at present several methodologies have been developed to assess the contribution of each variable to the prediction process. For deeper elucidations, Olden et al. [13] provided a comprehensive review and comparison of these methodologies.

In particular, sensitivity analysis is the term used to define a collection of methods that evaluate how sensitive model output is to changes in the values of predictive variables [14]. In ecology, the main sensitivity analysis methods applied to ANNs can be classified into four categories: (i) the Lek’s profiles method [15], [16]; (ii) the Perturbation method [17], [18]; (iii) the Partial Derivatives method [19], [20], [21], [22]; (iv) the Weights method, developed by Garson [23] and then implemented by Olden & Jackson [24]. Lek’s profiles study each input variable by keeping all other parameters at fixed values, while in Perturbation method each input variable is perturbed according to empirically established ranges while all others are kept untouched. The Partial Derivatives method involves small changes in each input variable and the evaluation of their relative contribution by computing the partial derivatives of the ANN output with respect to changes in the input. In the Weights method the connection weights of the ANN model are partitioned to evaluate the relative importance of each input variable and its positive or negative contribution to the model output. In the application of the first three methods, the values assigned to input variables can be devoid of real ecological meaning, i.e. they can be out of the range that is likely to be observed in real-world conditions. In these cases, environmental variables are forced to values that are only aimed at evaluating the model output, with no attention to the actual probability of recording those values given the (fixed) values of all the other variables. In fact, while of course the above-mentioned methods may provide valuable information about the way the “black-box” model works, the role of ecological relationships in constraining the multidimensional space where meaningful data patterns exist is not fully taken into account. With regard to the Weights method instead, the estimation of the input variables importance based on the connection weights may result unbalanced in certain cases where *constrained* training procedure may be applied to the ANN model for optimization purposes [25] (NB: in this sentence the term *constrained* is referred to the training procedure developed by Scardi [25] and it has nothing to do with the constrained perturbation of input variables here illustrated).

Therefore, although all those methodologies proved to be means of determining the overall numerical influence of each predictor variable to the model output, approaches that only consider changes consistent with the ecological relationships among environmental variables have never been proposed. It is well known in ecology that most environmental variables are far from independent of each other [26], [27]and therefore not all the combinations of their values are likely to occur (e.g. river slope tends to increase with elevation, as does the water oxygen concentration, and cannot be very steep in a floodplain). As these relationships constrain each variable in the complex multidimensional space that represents the abiotic conditions found in an ecosystem, some combinations of values are more easily found, while others just cannot occur. In fact, for instance, it would be highly unlikely for the maximum width of a stream channel to occur in a headwaters reach.

These issues raise the question: what is the point of perturbing or fixing variables at values which are ecologically meaningless? Evaluating the model output response in areas of the multidimensional space where environmental descriptors take far-fetched values may not be useful from an ecological perspective. Indeed it would make more sense to evaluate how sensitive model output is to changes in predictive variables values taking into account only plausible perturbations, i.e. changes which are consistent with the ecological relationships between environmental variables.

This study demonstrates an example of a new type of sensitivity analysis, using a case study about an ANN model aimed at predicting fish species richness in central Italian rivers. The goal of this work is to evaluate the real contribution of each predictive variable to species richness estimates by taking into full account the underlying ecological relationships and constraints. This way, all the perturbations applied to predictive variables reflect plausible environmental conditions, thus evaluating shifts in fish species richness only among ecological meaningful scenarios.

## 2. Material and methods

### 2.1. Study area and data collection

**Fig 1:**
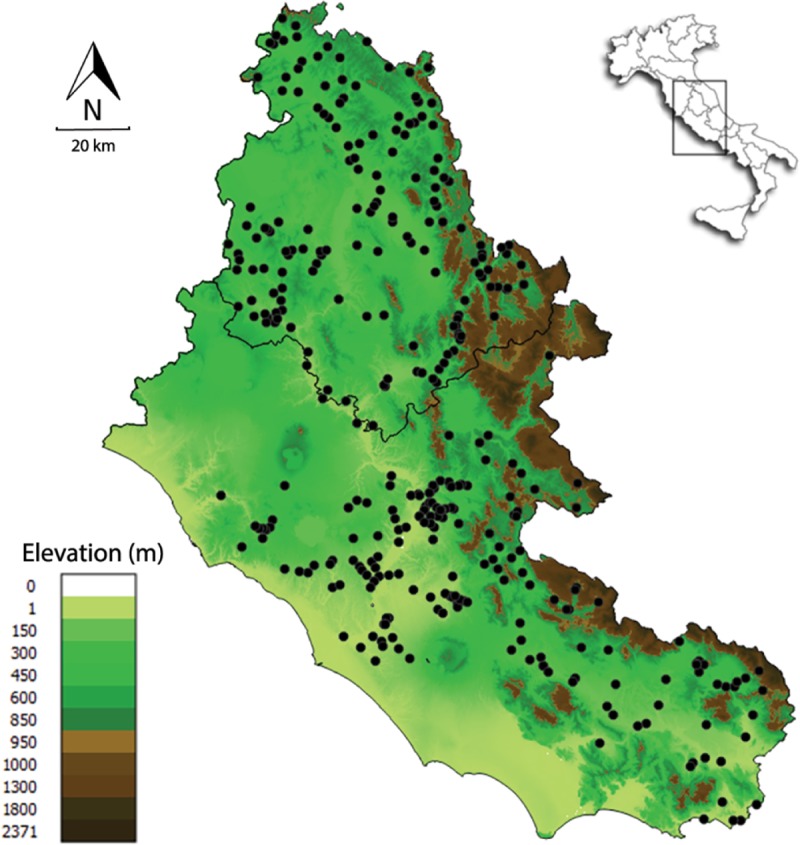
Sampling sites. Elevation map of the river basins of latium and umbria administrative regions in central Italy. Black dots mark the position of sampling sites. The image was obtained by using QGIS 2.18 (http://www.qgis.org).

Data have been obtained from 368 sites that have been sampled from 2009 to 2014 in central Italy [28], [29] (Fig 1). Most rivers in this area are characterized by a Mediterranean climate, hydrological regimes affected by rainfall variability and strong seasonal discharge variation, with high flows in spring and fall, and droughts in summer [30].

Fish sampling and environmental data acquisition were carried out according to the official Italian sampling protocol [31]. It generally consists of electrofishing sampling using a standard electro-fish shoulder-bag (4KW, 0.3–6 Ampere, 150–600 Volt). All available habitats were sampled along a stream channel 40–70 m long (the transect length was about 20 times the width of the wetted channel). Field activities were carried out beyond parks or protected areas. No endangered or protected species were involved and no specimen were harmed during the study nor collected. The occurrence of 55 fish species and values for 27 environmental variables (Table 1) were recorded at each site during sampling activities. Most of these variables had been already considered in previous studies [9], [32], [33].

**Table 1:** Environmental variables used as input to the ANN model. All environmental data have been obtained according to the official Italian sampling protocol [31].

Variable	Label	Min	Max	Mean	Median
**Slope (%)**	**SLP**	0	23.4	1.46	0.83
**Channel width (m)**	**CHW**	0.8	20	6.04	4
**Elevation (m)**	**ELV**	0	973	236.61	212.5
**Depth (m)**	**DET**	0.05	20	0.49	0,35
**Runs (area %)**	**RUN**	0	100	50.78	50
**Pools (area %)**	**POL**	0	100	23.99	20
**Riffles (area %)**	**RIF**	0	100	24.32	15
**Wetlands (0/1)**	**WEL**	0	1	0.07	0
**Bars & islands (0/1)**	**BAS**	0	1	0.05	0
**Boulders (area %)**	**BOL**	0	70	9.86	0
**Rocks & pebbles (area %)**	**ROK**	0	80	30.38	30
**Gravel (area %)**	**GRV**	0	90	24.47	20
**Sand (area %)**	**SAD**	0	80	20.20	20
**Silt & clay (area %)**	**SIT**	0	100	15.17	0
**Velocity (0–5)**	**VEO**	0	5	1.89	2
**Vegetation cover (area %)**	**VEC**	0	90	13.84	10
**Shade (area %)**	**SHD**	0	90	41.2	40
**Anthropic disturbance (0–4)**	**AND**	0	4	2.36	2
**Upstream barrier (Km, 0–100, 100 if no barrier)**	**UPB**	0.01	100	62.41	100
**Downstream barrier (0/1)**	**DOB**	0	1	0.51	1
**Upstream lake (Km, 0–50, 50 if no lake)**	**UPL**	0.2	50	45.85	50
**Temperature (°C)**	**TEP**	2.56	28.3	14.89	14.65
**pH**	**PHP**	4.88	9.45	8.04	8.09
**Conductivity (mS/cm)**	**COD**	229.2	1659	639.02	594.5
**O2 (%)**	**O2O**	7.31	160	88.76	93.08
**Source distance (km)**	**SOD**	0.01	233	21.49	11.99
**Sampled area (m**^**2**^**)**	**SAA**	30	3500	530.35	400

Channel width was always less than 20 m, since sample sites were primarily located within foothills and mountain zones. Thus, sampling methods (electrofishing) was standardized across sites, where wider river widths would have required nets or other gears.

### 2.2. Data set processing

All quantitative or semi-quantitative environmental data were normalized in the [0, 1], interval while qualitative data (e.g. wetlands or islands presence) were coded as binary values (0–1). Data normalization is a common procedure in ANNs model development [16], [17], since it transposes the predictive input variables into the data range on which sigmoid activation functions are based, thereby helping to approach to global minima at the error surface. As very steep slopes were only observed at two sites (13.4% and 23.4% respectively), slope data were normalized, omitting these two values, relative to third steepest slope value (9%). The maximum normalized value, i.e. 1, was assigned these outliers after normalization. This solution was adopted to prevent the compression of the normalized slope values into a very narrow range because of a couple of cases that cannot be regarded as part of a continuum. Species richness values were also normalized in the [0, 1] interval.

The whole data set was divided into three subsets (i.e. training, validation and test). The training set included 50% of records, while validation and test set included both 25% of records. Records were assigned to each subset by sorting all data according to ascending values of fish species richness and by dividing the resulting ordered sequence into groups of four records. Then the first and third record in each group of four records were assigned to the training set, while the second assigned to the validation test and the fourth to the test set. This procedure allowed to avoid unbalanced levels of species richness in the three data subsets.

### 2.3. Artificial neural network modeling

In this study, a three-layered feedforward network with bias has been trained in order to predict species richness. The optimal number of neurons in the hidden layer was determined by comparing the performance of different networks with 1 to 30 hidden neurons. A sigmoid transfer function was used both for hidden and output layers, thus enabling the network to learn non-linear relationships between input and output vectors [34]. Mean Square Error (MSE) was computed for the validation set to quantify the goodness of fit of the ANNs during training. The training procedure was terminated as soon as the MSE stopped decreasing monotonically, thus preventing the overtraining of the model during the learning process. This approach favors better generalization of ANN models while predicting new cases, as previously described in several ecological papers [25], [26]. Several values of learning rate and momentum (range 0.1–0.5) were tested to optimize learning performances. ANNs training and testing were performed in R environment [35] by using the functions of the package h2o [36].

### 2.4. Constrained sensitivity analysis

In order to use a sensitivity analysis aimed at perturbing environmental predictive variables in an ecologically sound perspective, the dependencies between all environmental variables were first investigated.

In particular, for each *j*^*th*^ environmental variable, the following steps were performed:

■ A Euclidean distance matrix was computed between the test set observations taking into account all the environmental variables but excluding the *j*^*th*^ variable.
■ For the *i*^*th*^ observation, neighboring observations were selected by taking those within the first quartile of the (d_max_−d_min_) distribution, where d_max_ and d_min_ were respectively the maximum and the minimum distance between the *i*^*th*^ observation and all other observations.
■ The minimum (*j*_*min*_) and maximum (*j*_*max*_) values of the *j*^*th*^ environmental variable were selected within the neighboring, i.e. most similar, observations. This defined the range of values that the *j*^*th*^ variable can take for the *i*^*th*^ observation.
■ The *j*^*th*^ variable was perturbed in the [*j*_*min*,_ *j*_*max*_] range while all other variables were kept untouched.
■ Five perturbed values in the [*j*_*min*,_ *j*_*max*_] range for each predictive variable were then passed to the data pattern fed to the ANN model, whose output was compared to the target (i.e. observed) fish species richness.
■ The same process was iterated for each observation (i.e. for each sampling site in the test set).

The results of this constrained sensitivity analysis were then compared to those obtained from simple input perturbation, i.e. by adding white noise in the [-0.5, 0.5] range to each input variable while keeping all the others untouched.

The method was entirely implemented in R programming language [35]. An example code is provided in the S1 File.

## 3. Results and discussion

### 3.1. Artificial neural network model

**Fig 2:**
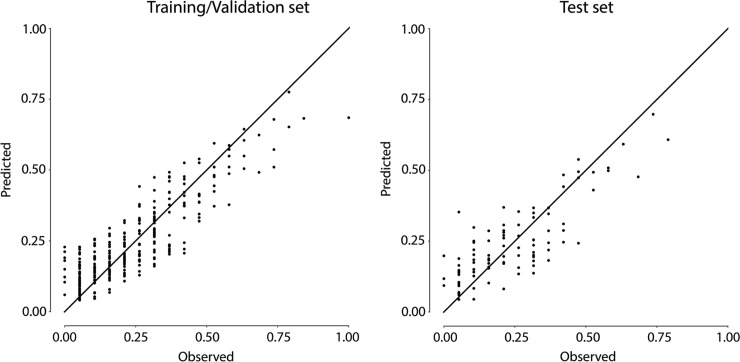
Predicted vs. observed species richness. Values on axes refer to normalized species richness. The determination coefficient for the ANN model was *R*^*2*^ = 0.771 for the training/validation set and *R*^*2*^ = 0.675 for the test set.

The best ANN architecture for predicting fish species richness on the basis of our environmental predictive variables had 8 hidden neurons and therefore a 27-8-1 structure. It explained a fairly large share of variance, ranging from *R*^*2*^ = 0.771 for the training/validation set to *R*^*2*^ = 0.675 for the test set (Fig 2).

The MSE (obtained from normalized data) varied correspondingly: MSE = 0.00756 for the training/validation set and MSE = 0.01001 for the test set. It seems that very low observed values of species richness are hardly reproduced by the model, possibly because the absence of species that could have been found on the basis of their ecological niche might depend on other factors (e.g. pressures not described by the available environmental variables) in species-poor situations. On the contrary, the highest values in the training set are slightly underestimated, while they match the observed values in the test set. However, the overall agreement between observed and predicted values is quite good with both data sets and is comparable to the level obtained in similar cases [12], [37], [38].

The average residuals relative to the normalized training data set as well as those relative to the normalized test set were very small (0.0017 and 0.0016, respectively), thus showing that the model was not systematically biased. In fact, when compared to the test set data, model predictions about species richness differed in only one species in 46% of the cases.

Although all the levels of species richness were included in both training and test data set, the model was less accurate when the highest species richness values were involved. This effect was most likely related to the difficulty of the ANN in identifying less frequent patterns (those with high species richness in this case), as already evidenced by Ozesmi et al. [39], thereby more easily leading to incorrect estimations. In fact, species richness values higher than 11 (normalized value = 0.631) were not frequently found, amounting to less than 5% of the whole data set.

### 3.2. Sensitivity analysis

#### 3.2.1. Constrained perturbations

All the methods for analyzing the sensitivity of ANNs relative to predictive variables are based on the assessment of changes in output values obtained as a consequence of known changes in input values. The procedure we present here has been implemented by constraining the random permutation method [17], [18], but its rationale (i.e. the same constraints) can be applied to any other method [21], [24].

**Fig 3:**
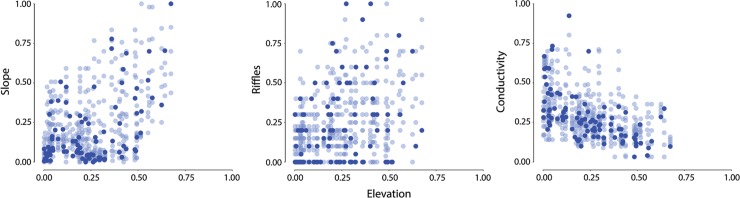
Constrained perturbations for slope, riffles and conductivity vs. elevation values. Perturbed values were obtained by applying the procedure outlined in section 2.4. The effect of the constraint is more evident for Slope and Conductivity, given their stronger dependence from elevation, than for Riffles, where it only limits the variability at low Elevation. Both observed (dark dots) and perturbed (lighter dots) values are shown.

In order to outline the differences between the way input data are perturbed by any unconstrained procedure and the way they are by our constrained approach, Fig 3 shows observed (dark circle) and perturbed (light circle) values for three environmental variables (Slope, Riffles and Conductivity) in scatter plots against elevation. Elevation is obviously not independent of some environmental variables and constrains their values according to the procedure outlined in section 2.4. In particular, in this example, constrained ranges are clearly visible on slope (positively correlated to elevation) and conductivity (negatively correlated to elevation), while perturbations of riffles values are very close to the maximum potential range in the ether upper quartiles of the elevation range, as a consequence of a much looser dependence of this variable from elevation.

The effect of random perturbations of slope and conductivity (i.e. complete independence between variables) would have been to fill up all graphs, while points representing perturbations of the two environmental variables occupy only a portion of the two-dimensional space, thus showing that some combinations of values are very unlikely to be observed. Perturbations showed in Fig 3 consist only of values that are more likely to be found in real-world conditions, although their range is large enough to allow assessing their impact on model behavior. Fig 3 is obviously depicting a very simplified set of relationships (only 3 out of 27 predictive variables). In practice, however, the same concept was applied to an *n*-dimensional space, where *n* is the number of environmental predictive variables used for the model development, thus defining an *n*-dimensional envelope that constrains the random perturbation of each environmental variable, excluding very unlikely patterns (e.g. very steep slope at very low elevation) from the sensitivity analysis.

#### 3.2.2. MSE percentages differences

**Fig 4:**
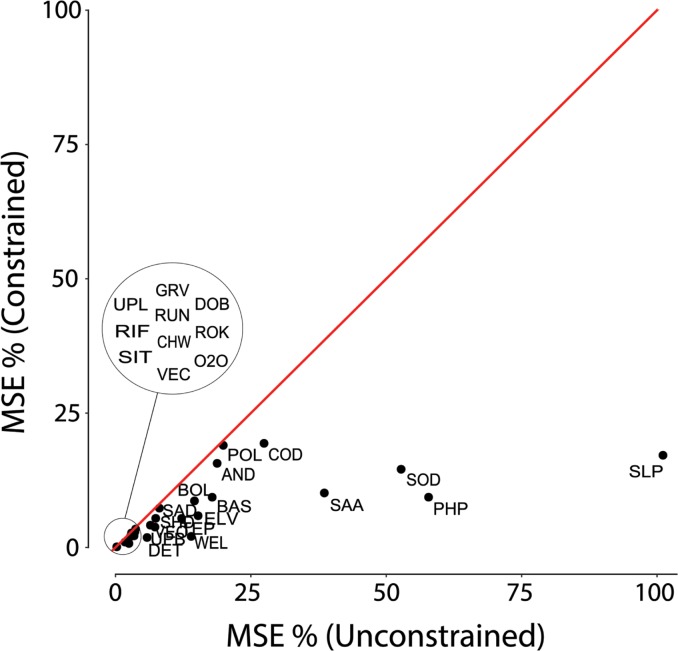
Percent increase in MSE obtained by constrained vs. unconstrained perturbations. Constrained sensitivity analysis clearly reduces maximum perturbations for the environmental variables, thus resulting in smaller increases in MSE for all of them (all points are below the unit slope line). However, the effect of the constraint is larger for some variables (e.g. Slope, SLP; pH, PHP; Source distance, SOD; Sampled area, SAA). See Table 1 for the names of environmental variables corresponding to other point labels.

The percent increase in MSE obtained by constrained perturbation of each variable for the test set is shown in Fig 4 versus the percent increase obtained by unconstrained perturbation. Unconstrained perturbations obviously induce larger increases in MSE, as they modify known data patterns to a larger extent. Although ANNs may respond to changes in a single input variable in a non-monotonic way, thus potentially making a large change in an input value less influential than a smaller one, in practice larger changes in input variables are clearly associated with larger increases in MSE. However, very large increases in MSE obtained from data patterns that are unlikely to occur in practical applications of the model are not useful–and possibly misleading–when it comes to the very purpose of sensitivity analysis, i.e. at inferring the role each input variable plays relative to the target variable.

While all the input variables are more sensitive to unconstrained perturbations, some show negligible differences between the two perturbation strategies, while others exhibit sharp differences. According to changes in MSE, the input variables that showed largest differences between the two perturbation methods were Slope (101.1% and 17.1%, for unconstrained and constrained perturbations, respectively), pH (57.8%; 9.3%), Source distance (52.7%; 14.5%) and Sampled area (38.5%; 10.1%).

Variables whose perturbations affected the model to a very limited extent (less than 10% increase in MSE), i.e. those in the lower left corner of Fig 4, do not deserve any further comment, because they certainly play a less important role. Other variables, however, are associated with changes in MSE between 10% and 30% and their constrained perturbation in some cases (e.g. Conductivity, Pools and Anthropic disturbance) induces changes in MSE almost as large as unconstrained and even more than the constrained perturbation of the “most influential” unconstrained (Slope, pH, Source distance and Sampled area).

In ecology, it is well known that fish species composition in lotic ecosystems tends to follow a typical longitudinal pattern [4] (i.e. differences in fish guilds occurrences and abundances) and generally fish species richness generally tends to increase with the distance from the river source [40]. Of course, there are field conditions that can be regarded as exceptions to this general trend. In fact, habitat features [41], [42], hydrological factors [43] or urbanization [44] may highly affect fish species diversity. It is clear that environmental variables like slope, pH or distance from source may provide information about the riverine trait where a site to be modeled is located (e.g. mountain or hilly region) [45], thereby providing valuable input information to the ANN model about expected species richness and inducing large changes in MSE when their values are perturbed. However, unconstrained perturbations, especially with those variables, may result in combinations of values, e.g. a steep slope too far from the source, that are unlikely or even impossible to occur in real-world situations, but that could trigger large changes in MSE.

Sensitivity analysis based on unconstrained perturbations can be deeply affected by this problem and the reason is that any model (and ANNs are no exception) is fitted to known data patterns, which obviously include only the combination of input values that actually occur in real-world situations. Extreme values may occur, but only in combination with a narrow range of values for other variables. Moreover, environmental variables are often strongly correlated with each other and their correlations make the range of ecologically meaningful variation in their values even narrower. For instance, pH usually decreases as the distance from river source increases, while conductivity increases [46]. These relationships make perturbations for Slope, pH, Sampled area, Source distance and Elevation strictly related to the ecological context, thereby defining a narrower, but more realistic range of values that can be safely used in practical applications of the model. Therefore, the MSE increase associated to large perturbations of these variables has very little importance relative to real world applications of the model.

#### 3.2.3. Importance of the environmental variables

**Fig 5:**
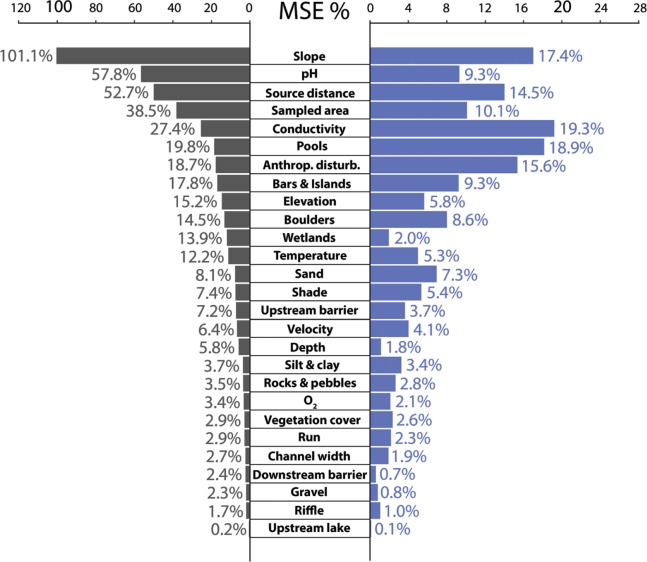
Unconstrained and constrained sensitivity analysis compared. Bars show the percent increase in MSE caused by the perturbation of each variable. Black bars (left) are for unconstrained perturbations while blue bars (right) are for constrained ones. Environmental variables are ordered according to the rank of their importance in the unconstrained sensitivity analysis. As the increase in percent MSE was smaller in constrained sensitivity analysis, the MSE axis was scaled accordingly to better show the relative length of the bars.

Changes in MSE after perturbation of each environmental variable were sorted in decreasing order after the application of a conventional scheme for sensitivity analysis and after the application of the constrained procedure. The outcome relative to the unconstrained procedure can be regarded as a different and simplified view relative to Fig 4. In fact, the bar diagram in Fig 5, just shows the increase in MSE caused by the perturbation of each variable. On the left after unconstrained perturbation and on the right after constrained perturbation. MSE% scales show the percent increase in MSE and are different in the two cases, as constrained perturbation cannot induce a level of increase in MSE as large as that induced by unconstrained perturbations.

In fact, all variables were obviously associated with smaller changes in MSE when the constrained procedure for sensitivity analysis was applied and the largest differences in the rank of variable importance occurred for Slope, Conductivity, pH, Sampled area, Pools and Anthropic disturbance, while less important environmental variables showed only minor shifts in their relative importance. pH was one of the most important variables according to the conventional procedure of sensitivity analysis based on unconstrained variable perturbation, but it only ranked eighth in sensitivity analysis based on constrained perturbations. Similar downgrades in importance were also observed for Slope and Sampled area. They are not surprising, as they occurred because of the narrower range of perturbed values these variables can assume under the constrained procedure for sensitivity analysis. In fact, this procedure takes only into account an amount of variability that is consistent with the observed relationships between variables and with the environmental context of each data pattern. As a consequence, environmental variables that had an intermediate relative importance according to the unconstrained procedure (e.g. Conductivity, Pools and Anthropic disturbance), gained a more relevant role as potential drivers of the local fish species richness.

While this result cannot be formally validated, as the true relative importance of the environmental variables is obviously unknown, it demonstrated an important feature of the constrained sensitivity analysis. The unconstrained procedure suggested a ranking of variables importance that showed what made the ANN model learn to recognize the riverine trait where sampling sites are located, thus obtaining estimates for fish species richness. However, species richness was also affected by variables that convey information about some relevant local conditions, like habitat features, hydrologic factors or urbanization. As a matter of fact, several studies evidenced that, at local scale, urbanization and/or flow regulation may strongly modify the expected fish species richness [40], [47]. Results obtained from the constrained sensitivity analysis showed indeed how, at any given site, fish species diversity is highly affected by environmental factors as habitat descriptors (e.g. Pools; Bars & islands) and anthropic disturbance (Conductivity; Anthropic disturbance). As conductivity can be considered as an indirect measure of water pollution [48], [49] and anthropic disturbance in most cases is related to urbanization, it is reasonable that they had a strong impact on fish assemblage diversity and composition.

In this work we focused on the estimation of variables importance taking into account first-order effects, as one input variable at a time was perturbed, while all other variables were kept untouched. Estimating the model output response to two-way [22] or more complex interactions between variables is certainly feasible in a constrained sensitivity analysis, but the problems related to the complexity of the procedure remain unsolved, making the analysis of higher order interactions between predictive variables practical only when their number is very small.

A very common goal in ANN modeling is the reduction of the number of input variables. The reason for that reduction is twofold: it might reduce the cost of predictive information and it might help to fight the *curse of dimensionality* [50]. The first problem depends on the way predictive information is collected: if all predictive data are already available, or if they are collected with no additional costs, e.g. during the same field activities, then the overall cost of predictive information will not be affected. The second problem is strictly related to the ratio between the number of available records and the number of input variables. According to Theodoridis & Koutroumbas [51], acceptable values for that ratio are in the 2 to 10 range, with smaller values that might result in a reduced prediction ability of the model.

As our data set was already available and all the predictive variables are routinely included in monitoring activities, no reduction in the cost of information could be achieved. Moreover, the number of available records (N = 368) is quite large relative to the number of predictive variables (p = 27) and therefore the ratio between the two (N/p = 13.63) is even larger than the upper limit of the above-mentioned range. Therefore, reducing the number of input variables was not needed, while preserving the full set allowed testing the constrained sensitivity analysis on a wider spectrum of variables. Moreover, preserving the full set of input variables allowed to exploit all the potential high-order relationships between variables that a trained ANN is able to capture and embed in its synaptic weights.

However, selecting the most important variables on the basis of a sensitivity analysis can be needed in data-limited scenarios and therefore we checked the effects of a reduced set of input variables, selected through a constrained sensitivity analysis, on the performance of the resulting ANN model. A subset of input variables was selected, including only those whose constrained perturbation induced increases in MSE larger than 10% (Fig 5), i.e. conductivity, pools, slope, anthropic disturbance, source distance and sampled area. Then a new ANN model with a 6-4-1 structure was trained and the determination coefficient for the test set was *R*^*2*^ = 0.44. Even if model accuracy in predicting fish species richness values considerably decreased, the variance explained by the model using the selected variables was still acceptable, especially in the light of the exclusion of 21 variables out of 27.

As far as we know, problems related to the scaling of ANN input variables (e.g. because of heterogeneity in their units) have been already tackled [52], [53], but methods aimed at defining to what an extent normalized input variable can be perturbed or changed in a sensitivity analysis, while preserving reasonable quantitative relationships with each other have never been implemented. From an ecological point of view, the method we propose showed what environmental variables, in real-world conditions (i.e. with values that vary within a realistic range) may actually induce changes in fish species richness. Looking at the results from a conservation perspective, assigning the highest degree of importance to variables that are very unlikely to change at local scale (e.g. slope) would be meaningless, while considering as more influential variables that may have a real impact on the fish assemblage richness, such as the level of water pollution or alterations of river traits due to urbanization [54], [55] is certainly more appropriate.

## 4. Conclusions

While several methods are available to test the sensitivity of ANNs or of any other type of model, we based our analysis on the perturbation method, because it is the one that most closely matches the rationale of the procedure we propose. However, the same rationale may be adapted to any other method (e.g. Partial Derivatives or Lek’s profiles method), as its only goal is to avoid data patterns that are not likely to occur in real-world conditions and that therefore are not really useful to open the ANN “black-box” as well as any other type of empirical model and to elucidate the way it worked and the ecological relationships it captured.

Of course, it was not possible to validate the approach we proposed by means of statistical analyses or by any other method. However, it showed that variables that influence fish species richness according to a procedure that takes into account only combinations of values that are likely to occur in real-world situations are not the same that would have been selected according to a procedure that does not take the ecological relationships between environmental variables into due account. Thus, our constrained approach to sensitivity analysis can be regarded as more realistic way to look into the model behavior, focusing on a meaningful subset of the multidimensional space in which the model can be theoretically applied. In fact, investigating how a model behaves in a region of its potential input space that will never be used in practical applications seems definitely pointless.

Needless to say, the procedure we proposed is only aimed at demonstrating a concept and therefore further developments can be imagined in its future applications, particularly as regards the selection of the number of neighboring observations or the maximum distance to them, thus investigating the effect of different levels of constrained perturbations and their effects in the resulting ranking of environmental variables importance.

## Supporting information

S1 File Constrained sensitivity analysis algorithm. Here, the R code algorithm of the constrained sensitivity analysis is provided.
(R) Click here for additional data file.

S2 File Data set. Data set used for the Artificial Neural Network modeling. All values were normalized as described in the Material and Methods section.
(CSV)
